# Pre and Probiotics Involved in the Modulation of Oral Bacterial Species: New Therapeutic Leads in Mental Disorders?

**DOI:** 10.3390/microorganisms9071450

**Published:** 2021-07-06

**Authors:** Yoann Maitre, Rachid Mahalli, Pierre Micheneau, Alexis Delpierre, Marie Guerin, Gilles Amador, Frédéric Denis

**Affiliations:** 1 Emergency Department, Montpellier University Hospital, 2415 Montpellier, France; maitreyoann@yahoo.fr; 2 Aide à la Décision pour une Médecine Personnalisée, Université de Montpellier, 2415 Montpellier, France; 3 Department of Odontology, Tours University Hospital, 7505 Tours, France; rachid.mahalli@etu.univ-tours.fr (R.M.); pierre45892@gmail.com (P.M.); alexis.delpierre@outlook.fr (A.D.); 4 Faculty of Dentistry, Clermont-Ferrand University, 63000 Clermont-Ferrand, France; marie.guerin@etu.uca.fr; 5 Faculty of Dentistry, Nantes University, 44035 Nantes, France; gilles.amador@univ-nantes.fr; 6 Faculté de Médecine, Education, Ethique, Santé, Université François-Rabelais, 7505 Tours, France

**Keywords:** mental health, oral microbiota, mental disorders, prebiotics, probiotics

## Abstract

This systematic review aims to identify probiotics and prebiotics for modulating oral bacterial species associated with mental disorders. Using the Preferred Reporting Items for Systematic Reviews and Meta-Analysis guideline, we search the electronic MEDLINE database published till January 2021 to identify the studies on probiotics and/or prebiotics for preventing and treating major oral dysbiosis that provokes mental disorders. The outcome of the search produces 374 records. After excluding non-relevant studies, 38 papers were included in the present review. While many studies suggest the potential effects of the oral microbiota on the biochemical signalling events between the oral microbiota and central nervous system, our review highlights the limited development concerning the use of prebiotics and/or probiotics in modulating oral dysbiosis potentially involved in the development of mental disorders. However, the collected studies confirm prebiotics and/or probiotics interest for a global or targeted modulation of the oral microbiome in preventing or treating mental disorders. These outcomes also offer exciting prospects for improving the oral health of people with mental disorders in the future.

## 1. Introduction

The oral cavity is an important bacterial gateway that plays a crucial role in the first-step digestion. Food entering the mouth is chewed and mixed with saliva before being swallowed. Microorganisms from the air pass through the nose and mouth into the trachea and lungs, and colonize the oral cavity. They spread to the epithelial surfaces and the body via the bloodstream. Thus, oral cavity is colonized by 50 to 100 billion bacteria [1], and they are responsible for many infectious diseases in the mouth, which include caries (tooth decay) and periodontitis (gum disease) [2,3]. Evidence confirms that oral bacteria are linked to many systemic diseases [4], such as cardiovascular disease [5], stroke [6], premature birth [7], diabetes [8], pneumonia [9], cancer development [10], kidney diseases [11], and mental disorders [12].

In reverse, *Bifidobacterium dentium* in the oral cavity may offer health benefits to the host [13]. Benefits include the barrier against pathogens or immunomodulating properties [14]. Thus, many bacterial strains are identified as probiotics—microorganisms whose ingestion in adequate quantities is beneficial to the health of the host. According to the International Scientific Association for Probiotics and Prebiotics, Bifidobacterium (*adolescentis*, *animalis*, *bifidum*, *breve* and *longum*) and Lactobacillus (*acidophilus*, *casei*, *fermentum*, *gasseri*, *johnsonii*, *paracasei*, *plantarum*, *rhamnosus* and *salivarius*) represents a core group of well-studied species likely to impart some general benefits [15]. Similarly, prebiotics are substrates, such as fructans and galactans, selectively used by host microorganisms and offered a health benefit by modulating the microbiome of individuals [16].

**Figure 1:**
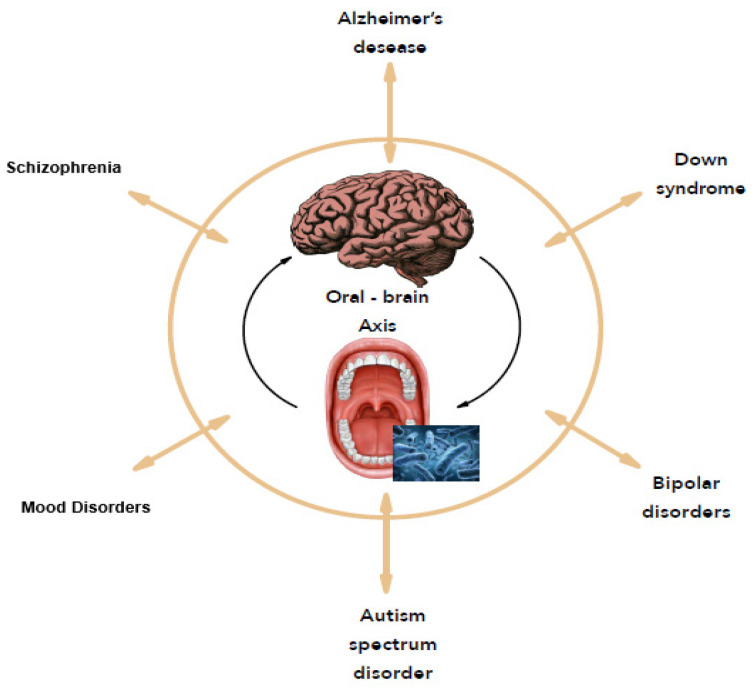
The oral-brain axis.

In 2001, Joshua Lederberg introduced the term “microbiome” [17] referred to as dynamic communities of microbes that colonized the body and provided many metabolic functions and molecular signals to maintain good health. With the concept of the microbiome, microbiologists have refocused on microbial communities rather than on individual organisms in pure culture, as in Koch’s postulates. In this case, the consortia of organisms in a biofilm rather than a single pathogen are responsible for many infections like caries, periodontitis, and others [18]. As many people never develop dental caries, some authors suggest that certain bacterial species have a potential antagonistic effect against cariogenic bacteria [19]. Thus, replacing pathogens with harmless isolates obtained from healthy strains prevent health disorders. A metagenomic approach confirms that the bacteria with a protective effect against cariogenic species offers probiotic benefits for the oral health of the host [20]. From an ever-growing understanding of how the microbiome affects health and disease, the human microbiome offers new therapeutic pathways like reversing or rebalancing the microbiome towards health. In this context, this study focuses on the oral microbiome and its correlation to mental disorders, named the “Oral-Brain Axis” [12] to highlight new therapeutic perspectives for mental disorders (Figure 1).

The identification of a specific microflora in patients with mental disorders suggests that these disorders could be influenced by the oral microbiome. For example, the inflammatory process induced from the Gram-negative periodontal pathogen *Porphyromonas gingivalis* is evoked in individuals with dementia or Alzheimer’s Disease [21,22]. In Autism Spectrum Disorder, the specific oral dysbiosis observed in the oral microbial community of these patients suggests a potential role for microorganisms in the progression of this disease [23]. The microflora of individuals with Down’s Syndrome (DS) with periodontitis show similarities with those of patients without DS with periodontitis, including the main periodontal pathogens (*Aggregatibacter actinomycetemcomitans*, *P. gingivalis*, *Tannarella forsythia*, *Prevotella intermedia*, *Parvimonas micra*, *Fusobacterium nucleatum*, *Campylobacter rectus*, etc.). In addition, patents with DS have increased colonization of some bacterial species in the subgingival flora (*Selenomonas noxia*, *Propionibacterium acnes*, *Streptococcus gordonii*, *Streptococcus mitis*, *Streptococcus oralis*, *Treponema socranskii* and *Streptococcus constellatus*). *S. gordonii*, *S. mitis*, and *S. oralis* initiate early microbial colonization and contribute to the development of microbial plaque. The presence of *P. acnes* (normally found on the skin) at high levels in the subgingival microbiota of subjects with DS may be related to the habit of putting fingers to the mouth [24]. *S. gordonii* contains genes that facilitate the attachment of free-floating *P. gingivalis* to the adherent plaque biofilm [25]. *S. noxia* is associated with periodontal disease activity at interproximal sites [26] and *T. socranskii* has been associated with the severity of periodontal tissue destruction [27]. Finally, *S. constellatus* is associated with refractory forms of periodontitis [28]. Although this overall periodontal dysbiosis may be influenced by oral hygiene habits and deficient immune status of DS patients, it could contribute to an increased susceptibility to periodontitis in patients with DS [29]. Bipolar disorder was correlated with an increased risk of periodontitis, a higher frequency of periodontopathogens, and a higher total bacterial load like *A. actinomycetemcomitans* and *P. gingivalis* [30]. Finally, the oropharynx microbiome in schizophrenics is significantly different from that of healthy controls, with some bacterial species more abundant in schizophrenic patients than in controls (*Lactobacillus gasseri*, *Lactobacillus salivarius*, *Bifidobacterium pseudocatenulatum*) [31].

**Figure 2:**
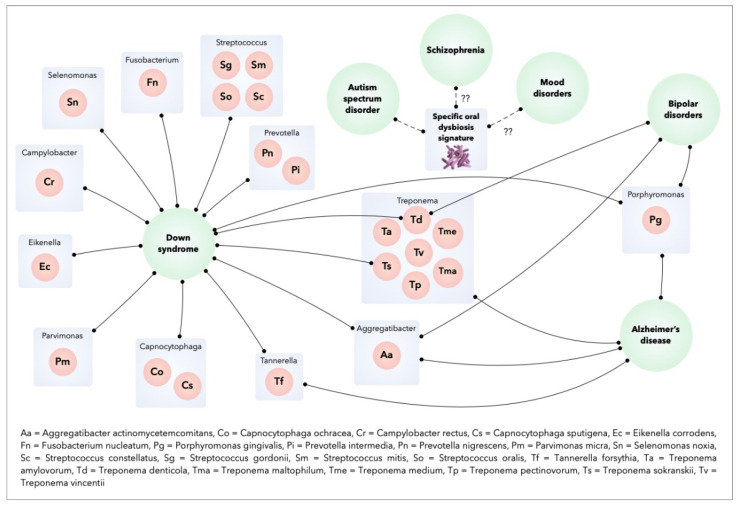
Oral bacteria and mental disorders.

Therefore, the concept of the “oral-brain axis” refers to the role of the oral microbiota in the biochemical signaling events between the oral microbiota and central nervous system [10]. The bacterial species associated with mental disorders through the “oral-brain axis” are mainly periodontopathogen bacteria (*A. actinomycetemcomitans*, *P. gingivalis*, *T. forsythia*, *Treponema denticola*, *C. rectus*, *P. intermedia*, etc.). Figure 2 presents the main bacterial species that may be potentially associated with mental disorders through the “oral-brain axis” [10,22,29,30,32,33].

Similar to the “gut–brain axis” that emphasizes the bidirectional communication between the central and enteric nervous systems [34], the possible relationship between the oral microbiome and mental disorders suggests that probiotics or prebiotics use could prevent and manage mental disorders by modulating oral dysbiosis and its inflammatory and immune consequences. Prebiotics are a type of fiber that the human body cannot digest. They serve as food for probiotics, which are microorganisms. Both prebiotics and probiotics may support helpful bacteria and other organisms in the gut, and we hypothesized in the oral cavity.

The objective of this systematic review is to identify probiotics and prebiotics involved in modulating oral bacterial species associated with mental disorders.

## 2. Materials and Methods

The present systematic review was conducted according to the PRISMA guidelines for Systematic Reviews [35].

### 2.1. Search Strategy

To identify studies that met the inclusion criteria, an electronic search was conducted through the MEDLINE (PubMed) database up to January 2021 without restriction. While modulation of the microbiome represents an innovative therapeutic axis to treat or prevent human pathologies [36], the impact of probiotics on human health has long been documented [37]. Consequently, the search was performed without time restriction to identify the studies that explore the application of probiotics and/or prebiotics in preventing and treating major oral dysbiosis potentially involved in mental disorders. The search terms were used in combination with Boolean operator “AND”/”OR” according to the following equations:

((“Probiotics”[Mesh]) OR “Prebiotics”[Mesh]) AND “Mouth/microbiology”[Mesh](1)

((“Probiotics”[Mesh]) OR “Prebiotics”[Mesh]) AND “Mouth Diseases/microbiology”[Mesh](2)

((“Probiotics”[Mesh]) OR “Prebiotics”[Mesh]) AND “Saliva/microbiology”[Mesh](3)

((“Probiotics”[Mesh]) OR “Prebiotics”[Mesh])) AND “Dental Plaque/microbiology”[Mesh](4)

### 2.2. Study Detection

A manual reference check of eligible studies on the subject was performed by two operators who reviewed the studies according to the inclusion/exclusion criteria.

### 2.3. Inclusion and Exclusion Criteria

The bacterial species associated with mental disorders (Alzheimer diseases, Autism Spectrum Disorders, Down Syndrome, and Bipolar Disorders) through the oral-brain axis are mainly periodontopathogen bacteria (*A. actinomycetemcomitans*, *P. gingivalis*, *T. forsythia*, *T. denticola*, *C. rectus*, *P. intermedia*, etc.). We included experimental or clinical studies (longitudinal, cross-sectional, or randomized) that identified the effects of probiotics strains or prebiotics compounds on these bacterial species or the host response associated. Conferences, abstracts, reviews, and editorials were excluded.

### 2.4. Data Collection

Two independent reviewers (M.G. and Y.M.) screened titles and abstracts to identify the relevant papers based on the inclusion criteria. The full studies were reviewed to decide whether they should be included when the abstract information was judged to be insufficient. The reviewers reached a consensus on the eligibility criteria for selecting the studies. In case of discrepancy, a third reviewer (F.D.) resolved the conflicts regarding the eligibility.

## 3. Results

### 3.1. Study Selection

**Figure 3:**
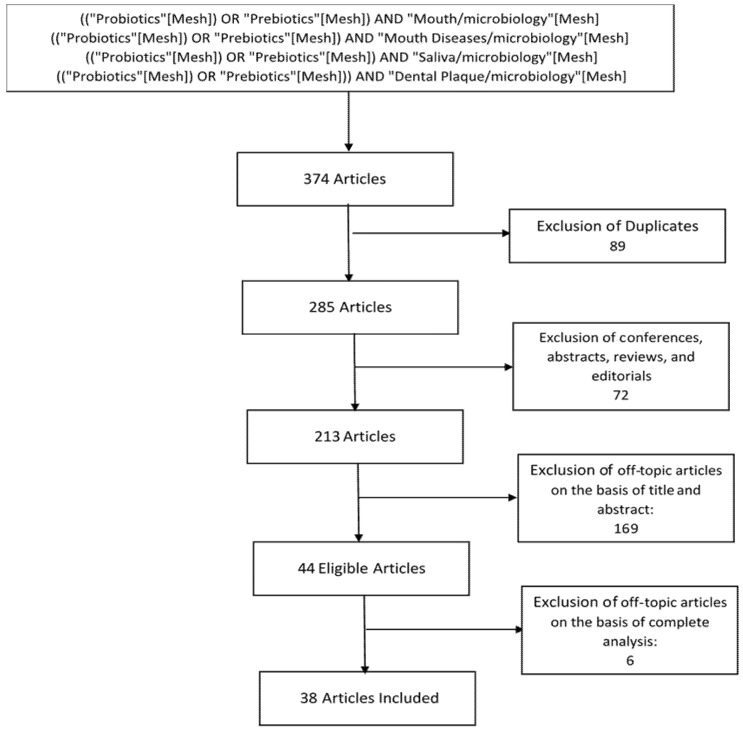
Flow chart of study selection.

The initial studies retrieved from the databases were first selected. We selected 38 studies from 285 studies (Figure 3), and we reviewed and analyzed studies that met the eligibility criteria.

Each study that met the inclusion criteria was analyzed to identify prebiotic compounds or probiotic strains and their effects on the oral microbial flora or immunological markers involved in mental disorders. Three distinct effects on oral dysbiosis associated with mental disorders emerged: modulation of the oral microbiome [38,39,40,41,42,43,44,45,46,47,48,49,50,51], antimicrobial activity [52,53,54,55,56,57,58,59,60,61,62,63,64,65,66,67,68,69], and modulation of immunological or inflammatory factors [43,44,46,56,70,71,72,73,74,75].

### 3.2. Prebiotics

From our search, three recent studies on prebiotics were identified [38,39,40] (Table 1). Each demonstrated an oral microbiome modulation. In an in vitro study, Slomka et al. [38] identified two bacterial nutritional compounds, beta-methyl-d-galactoside and *N*-acetyl-d-mannosamine, that induced a beneficial composition in dual-species biofilm communities (beneficial and pathogen). Beta-methyl-d-galactoside decreased *F. nucleatum* and *P. gingivalis* in the biofilm by stimulating *Streptococcus salivarius*. *N*-acetyl-d-mannosamine stimulated *S. mitis* and *Streptococcus sanguinis* resulting in a reduction of *A. actinomycetemcomitans* and *Streptococcus sobrinus*. Rosier et al. [39] demonstrated that in vitro exposure to the ecological factors such as Nitrate favored the flora associated with oral health (Genera Neisseria and Rothia). This is achieved by limiting periodontitis-associated genera (Porphyromonas, Fusobacterium, Leptotrichia, Prevotella, and Alloprevotella) without affecting biofilm growth. In 2019, Jiménez-Hernández et al. show that consumption of a mixture of bacterial growth substrates (short-chain galacto-oligosaccharides (5 g), long-chain fructo-oligosaccharides (10 g), and glutamine (5 g) for six weeks by 32 patients change the structure of the oral microbiota. This is characterized by a decrease in alpha diversity and a change in beta diversity, with no clear orientation towards a healthy microbiota [40].

### 3.3. Probiotics

In 35 studies published between 2008 and 2020, we identified the probiotics strains and their effects on the bacterial species potentially involved in mental disorders (Table 2).

Table 3 shows the different probiotic strains investigated alone or in combination in 12 in vitro studies [41,42,43,44,45,46,52,53,54,55,56,57,58,59,60] and 23 in vivo studies [46,47,48,49,50,51,59,60,61,62,63,64,65,66,67,68,69,70,71,72,73,74,75] including three using animal models [46,70,71]. The experiments in patients were performed with small samples (*n* = 12 to 108) and a short follow-up period (1 day to 36 weeks).

Various Galenic forms were explored in addition to the classic tablets and lozenges, such as milk drinks [48,50,63], yogurts [49], cheese [64], and mouthwashes [66,67].

#### 3.3.1. Modulation of the Oral Microbiome

The development and degradation of oral biofilms rely on adhesion and cooperation or competition between oral bacteria [76]. Several in vitro and in vivo studies demonstrated the ability of various probiotics strains to modulate these mechanisms. In vitro models, the *Lactobacillus reuteri* introduction results in changes to the nascent and developed biofilm ecosystem with an increase in exogenous lactobacilli (L.), streptococci (S.) and Gram-negative anaerobes [41]. *Lactobacillus rhamnosus* reduces the biofilm-forming capacity of *F. nucleatum*, integrates into all oral biofilms [42], and shows higher adhesive properties than *P. gingivalis* on gingival stromal cells [43].

Several probiotic strains (*L. reuteri*, *L. rhamnosus*, *Lactobacillus acidophilus*, *Lactobacillus casei*, *Bifidobacterium longum*, *Bifidobacterium animalis*, *Bifidobacterium breve*, *Bifidobacterium pseudolongum*, *Bifidobacterium bifidum*) can modulate the ability of *P. gingivalis* to adhere to and invade gingival epithelial cells [44]. The lipase enzyme from lactobacilli strains used as probiotics (*Lactobacillus acidophilus*, *Lactobacillus casei*) might be an influential factor in the biofilm degradation integrating *A. actinomycetemcomitans* [45].

In rats, Bifidobacterium reduces the proportion of some Gram-negative anaerobic bacteria-like species involved in the pathogenesis of periodontal diseases in the subgingival biofilm (*Veillonella parvula*, *Capnocytophaga sputigena*, *Eikenella corrodens*, and *Prevotella intermedia*) [46].

Similar results are found in several in vivo studies. Thus, *Bifidobacterium animalis* subsp. lactis HN019 used for 30 days (lozanges) reduce the adhesion of *P. gingivalis* to buccal epithelial cells [47]. Short-term consumption (1 day) of milk fermented with probiotic strains (*Streptococcus thermophilus*, *Lactobacillus delbrueckii*, and *Lactobacillus paracasei*) supplemented with vitamin B6 and vitamin D increases the overall diversity of the oral cavity microbiome with an increase in the genera Steptococcus and Actinomyces. However, no substantial change in the microbiome structure is noted [48,49]. The effects of *L. acidophilus*, *B. animalis*, and *L. rhamnosus* combined in a probiotic milk drink on periodontopathogenic bacteria in subgingival plaque is lower than those in supragingival plaque [50]. According to Tada et al., *L. reuteri* fails to improve the microbial flora of the peri-implant sulcus in patients with peri-implantitis [51]

#### 3.3.2. Antibacterial Activity

The antimicrobial activity of probiotics controls and inhibits the growth of certain microorganisms and bacteria [77]. In many vitro studies, human oral lactobacilli (*L. acidophilus*, *Lactobacillus crispatus*, *L. delbrueckii*, *Lactobacillus gasseri*, *Lactobacillus salivarius*, *L. paracasei*, *Lactobacillus plantarum*, *L. rhamnosus*, *Lactobacillus fermentum*, *L. caseï*) or Bifidobacterium (*B. animalis*) possesses an antimicrobial activity against oral pathogens. It also has a strong inhibitory effect against *S. mutans*, *S. sobrinus*, Gram-negative periodontal pathogens *P. gingivalis*, *A. actinomycetemcomitans*, *P. intermedia*, and *F. nucleatum* [52,53,54,55]. Shin et al. showed that *Lactococcus lactis* possesses the antimicrobial activity against *T. forsythia*, *T. denticola*, *F. nucleatum*, and *P. gingivalis* [56]. Higuchi et al. also observed *P. gingivalis* demonstrated a retarded growth when *L. salivarius* and green tea catechins were combined [57]. Zhu et al. confirmed that the probiotic strains (*L. delbrueckii*, *S.thermophilus*, *L. acidophilus*, *Bifidobacterium*) inhibited periodontal pathogens. However, no effect on the “protective bacteria”, *S. sanguinis*, was observed, and competition between probiotics and periodontal pathogens depended on the inoculation sequence [60]. According to Koll et al., *L. plantarum* is unsafe because it is different from the natural resistance pattern of lactobacilli [52].

In addition, this antimicrobial activity is observed in vivo. According to Invernici et al., *B. animalis* inhibits the growth of *P. gingivalis*, *P. intermedia*, *F. nucleatum*, and *A. actinomycetemcomitans* [47]. A decrease in the number of *P. gingivalis* was also reported after oral administration of *L. crispatus* for four weeks [59]. Administration of *L. reuteri* tablets for 28 days reduced the number of *P. intermedia* and *P. gingivalis* in the subgingival microbiota [60]. In combination with scaling and root planning (SRP), the consumption of *L. reuteri* tablets for 12 weeks showed a limited difference in the number of *P. gingivalis* [61]. The association between *L. plantarum, L. brevis*, and *Pediococcus acidilactici* demonstrated a significant microbiological impact after reducing the counts of *T. forsythia* in patients with gingivitis [62]. From Imran et al., daily consumption of *L. casei* commercial fermented milk (Yakult^©^) for one month reduced the numerical sum of *P. gingivalis*, *A. actinomycetemcomitans*, and *P. intermedia* in patients with chronic generalized mild to moderate periodontitis [63]. Consuming petit-suisse cheese for nine days, *L. casei* reduces the density of *A. actinomycetemcomitans* and maintain lowers density of *P. gingivalis* two weeks later [64]. Four weeks after intake of *L. salivarius* tablets for eight days, a significant reduction is noted in the number of *A. actinomycetemcomitans*, *P. intermedia*, *P. gingivalis*, *T. denticola*, and *T. forsythia* in the subgingival plaque. This reduction disappears after eight weeks [65]. Sajedinejad et al. found that oral application of *L. salivarius* as a mouthwash for 28 days serves as the antimicrobial activity against *A. actinomycetemcomitans* [66]. In a pilot study, a commercial probiotic mouthwash containing natural oral bacteria (*S. oralis*, *S. uberis*, *S. rattus*) shows a trend towards the reduction of periodontal pathogens in subgingival plaque (*P. gingivalis* and *C. rectus*) [67]. Daily consumption of probiotic lozenges that combined *L. rhamnosus* and *B. animalis* for four weeks decreases the bacterial load of *A. actinomycetemcomitans* and *F. nucleatum* in both saliva and plaque. The consumption also decreases the number of *P. gingivalis* in the plaque [68]. In patients who received non-surgical treatment (SRP), the administration of *L. rhamnosus* sachets (30 days) or azithromycin pills (five days) offered microbiological effects similar to SRP alone for the treatment of chronic periodontitis [69].

#### 3.3.3. Modulation of Immunological or Inflammatory Mediators of Oral Dysbiosis

The bacterial exposure induces the synthesis of cytokine as Interleukins (IL) or TNF α and chemokine as CXCL8. This synthesis regulates inflammation and modulates cellular activities associated with the host immune response [78]. Some probiotics demonstrated in vitro ability to modulate immunoinflammatory parameters. According to Shin et al., Lactococcus lactis neutralizes and inhibits the production of IL-6 or TNF-α induced by lipopolysaccharides from *F. nucleatum*, *P. gingivalis*, and *T. forsythia* [56]. CXCL8 secretions from gingival stromal stem cells increase when pretreated with *L. rhamnosus* before *P. gingivalis* stimulation [43]. In gingival epithelial cells, IL-1β and TNF-α synthesis stimulation decreases when co-culture with *P. gingivalis* and *L. rhamnosus* or bifidobacteria (*B. longum*, *B. animalis*, *B. pseudolongum*, *B. bifidum*) or *L. salivarius*. CXCL8 secretion increases when co-cultured with *P. gingivalis* and *L. salivarius* or *L. rhamnosus* [45]. In mice, *L. gasseri* and *L. brevis* decrease the expression and secretion in gingival tissue of inflammatory cytokine (IL-1β, IL-6, IL-17A, and TNF α) [70,71]. *B. lactis* reduces levels of IL-1b and IL-1b/IL-10 ratios in rats using experimental periodontitis [46]. These observations are different from those observed in humans. In a group of 47 individuals, 4-week consumption of tablets containing a mixture of *L. rhamnosus* and *L. curvatus* showed no effect on the concentration of selected cytokines (IL1β, IL6, IL8, IL10, TNF-α) in gingival crevicular fluid [72]. In two randomized, double-blind, placebo-controlled crossover trials, three-week ingestion of *L. reuteri* tablets twice a day offers no difference in cytokine saliva levels (IL1β, IL6, IL8, and IL10) [73,74]. Following the management of peri-implant mucositis, probiotic supplementation with *L. reuteri* offered no difference in inflammatory mediator level in the crevicular fluid for 89 patients at a 26-week follow-up [75].

## 4. Discussion

Many studies suggest beneficial effects of prebiotics and probiotics on brain function through the gut–brain axis [79]. In contrast, no studies demonstrate a use of prebiotics compounds or probiotics strains to prevent or treat brain disorders through oral-brain axis.

We identified 38 studies, and only three explored the effects of prebiotic compounds on the growth of beneficial bacteria. Prebiotics are a recent field of research [80], and scanty studies publish their effects on the oral microbiome. Our review shows that nutritional stimulation of the oral microbiome using various prebiotic compounds (Nitrate, beta-methyl-d-galactoside, *N*-acetyl-d-mannosamine, etc.) may induce the composition of the dental biofilm and growth of beneficial oral bacteria at the expense of pathogenic bacteria (*A. actinomycetemcomitans*, *F. nucleatum*, *P. gingivalis*). Anxiolytic and antidepressant effects or enhancement in cognitive deficit and social functioning were observed with the rebalancing of gut flora through the consumption of prebiotics by patients suffering from depression, Alzheimer’s disease, or autism spectrum disorders [81]. Thus, in the context of the oral-brain axis, it can be assumed that the use of prebiotics in modulating the oral microbiome could lead to improvements in mental health. As the beneficial effects of prebiotics have already been studied for the modulation of the gut microbiome [82,83], it can be assumed that research on the effects of prebiotics on the oral flora will represent an area with growing interest.

Since the 1980s, studies have explored probiotics, particularly lactobacilli of which the most popular are *L. rhamnosus*, *L. reuteri*, *L. casei*, and *L. acidophilus* [84]. Probiotic microorganisms that offer health benefits for humans are Lactobacilli and Bifidobacterium species [85]. The major probiotic mechanisms of the action of probiotics include improved adhesion to bacterial colonization sites, competing with pathogenic microorganisms, production of antimicrobial substances, and modulating the host’s immune response [86].

The in vitro and in vivo confirm the effects of lactobacilli and bifidobacterium on modulating the oral microbiota associated with mental disorders (*B. animalis*, *L. paracaseï*, *L. acidophilus*, *L. rhamnosus*, *L. delbrueckii*). Bacterial competition excludes some pathogens without biofilm structural disruption (*F. nucleatum*, *P. gingivalis*, etc.). The biofilm enzymatic degradation capacity appears as a mechanism of action implicated in this competition.

The specific dysbiotic signature associated with patients with mental disorders suggests an influence of the central nervous system in the development of oral pathologies [12]. While the use of probiotics may appear as a complementary therapeutic means for patients with mental disorders, it is necessary to keep in mind the multifactorial character of the oral microbiome homeostasis [87]. Frequent oral alterations affect patients with mental disorders [88]. These alterations are correlated with a psychomotor impairment that prevents an adequate hygiene routine and reduces salivary flow due to various psychoactive substances (drugs, medication) and difficulty in accessing dental health services [89,90]. These mental disorders promote oral dysbiosis that can lead to dental caries and periodontal disease [91].

*P. gingivalis*, *T. forsythia*, and *T. denticola* represent the red complex polymicrobial community involved in the development of periodontal disease [92]. Some of these periodontopathogen bacteria (*P. gingivalis*, *T. denticola*) or their toxic proteases (gingipaïn) detected by postmortem analysis of Alzheimer’s disease patients’ brains suggest their pathogenesis involvement of this mental disorder [93]. Thus, the antimicrobial activity of probiotic strains against periodontal pathogens could represent an axis of prevention of oral dysbiosis and its potential implication in the development of mental disorders.

The modulation of the immune system induced by prebiotics and probiotics is one of the health benefits of increasing interest. Although the mechanisms of action are not yet clearly understood, this stimulation of the immune system can be direct by altering cytokine expression or indirect by altering the composition and population of bacterial species. The direct beneficial effects on the immune system are generally associated with an increase in the expression of anti-inflammatory cytokines (IL 4, IL 10, IL 11, IL 13) and a reduction in pro-inflammatory cytokines (IL 1β, IL 6, TNFα) [94]. Their immunomodulatory effects have been extensively studied in inflammatory diseases in the gastrointestinal tract [95,96,97] but remain poorly evaluated in periodontal pathogen-induced inflammatory diseases.

Chronic inflammation and latent infections can cause cognitive and behavioral problems [98]. Cytokines produced outside the central nervous system such as IL 1β and TNF α cause brain neurotoxicity [78]. The inflammation induced by periodontopathogen bacteria is associated with dementia and neurodegenerative lesions in patients with Alzheimer’s disease [21,22]. In in vitro and animal studies, while probiotic strains seem to decrease in cytokines induced by the main periodontopathogens species (*P. gingivalis*, *F. nucleatum*, *T. forsythia*), the improvement of the inflammatory condition is not observed in human studies. The probiotics are not demonstrated in preventing the neurological consequences of inflammation associated with periodontal disease.

## 5. Limits and Perspectives

In this review, studies suggest that prebiotics and probiotics can prevent and treat oral dysbiosis involved in the oral-brain axis. However, several limitations are noted. These studies only use human small samples with short intake and/or follow-up, which fails to define their long-term effects. In addition, the diversity of probiotic species studied according to different modes of administration neither support the standardization of a probiotic formulation nor the definition of an adapted delivery system.

Prebiotics and/or probiotics are not shown to treat or prevent mental disorders using modulating oral dysbiosis. In addition, their effects are preventive approaches for periodontal disease. Human studies using different galenic forms (milk drinks, yogurts, and mouthwashes) should be explored in future studies for more suitable use of prebiotics and/or probiotics in patients with reduced oral hygiene habits [98]. Longitudinal studies should define a formulation of prebiotics and/or probiotics and a mode of administration in preventing oral dysbiosis and evaluating their safety.

## 6. Conclusions

Our review highlights the limited research regarding the use of prebiotics and/or probiotics in modulating oral dysbiosis in mental disorders. However, the studies confirm their interest in preventing or treating mental disorders through global or targeted modulation of the oral microbiome. Research is emerging on prebiotic compounds and probiotics for the treatment of oral dysbiosis and mental disorders. In this review, the probiotic strains belong to the genus Lactobacilli and Bifidobacterium commonly studied for the rebalancing of intestinal flora. While prebiotics and probiotics are part of the gut–brain axis, it is still difficult to envisage their preventive or therapeutic application for managing mental disorders through the oral-brain axis. Modulating oral dysbiosis can improve the oral health of patients with mental disorders.

## Figures and Tables

**Table 1:** Prebiotics: Summary of selected studies.

Reference(s)	Study Design	Prebiotic Compound	Objectives	Prebiotic Administration	Mains Results and Limitations *
Slomka et al. (2017) [38]	In vitro	m-inositol, lactitol, alpha-methyl-d-galactoside, beta-methyl-d-galactoside, turanose, *N*-acetyl-d-manno-samine, Met-Pro, Phe-Glu, l-aspartic acid andsuccinic acid.	Identification of potential oral prebiotics that selectively stimulate commensal albeit beneficial bacteria of the resident oral microbial community while suppressing the growth of pathogenic bacteria	Not relevant	Beta-methyl-d-galactoside and *N*- acetyl-d-mannosamine could be identified as potential oral prebiotic compounds, triggering selectively beneficial oral bacteria throughout the experiments and shifting dual species biofilm communities towards a beneficial dominating composition. Beta-methyl-d-galactoside selectively stimulated *S. salivarius* causing a decrease of *F. nucleatum* and of *P. gingivalis* in the biofilm. *N*-acetyl-d-mannosamine was the only compound that in all beneficial-pathogen combinations did not lead to an outgrowth of any of the pathogenic species. Besides, *S. mitis* and *S. sanguinis* were significantly stimulated causing a reduction of *A. actinomycetemcomitans* and S. sobrinus * in vitro, oral prebiotic compounds need to be confirmed in multi-species environments
Rosier et al. (2020) [39]	In vitro	Nitrate	Evaluation of the short-term effect of a single dose of nitrate on pH, oral biofilm growth and bacterial composition	Not relevant	Signifcantly higher levels of the oral health-associated nitrate (6.5 mM) -reducing genera Neisseria (3.1×) and Rothia (2.9×) were detected in the nitrate condition already after 5 h. Periodontitis-associated genera (Porphyromonas, Fusobacterium, Leptotrichia, Prevotella, and Alloprevotella) were significantly reduced after 5 h and 9 h. The addition of 6.5 mM nitrate did not show signifcant changes in real-time impedance measurements of bioflm formation compared to the control condition. * in vitro study
Jiménez-Hernández et al. (2019) [40]	Cross sectional study (*n* = 32)	Mixture of short-chain galacto-oligosaccharides, long-chain fructo-oligosaccharides and glutamine	Characterization of the compositional changes associated with prebiotic intervention on salivary microbiota in HIV-infected individuals. Study of the interplay between oral and gut microbiota determining the bacterial co-occurrences in both habitats.	Daily consumption of mixture of short-chain galacto-oligosaccharides (5 g), long-chain fructo-oligosaccharides (10 g), and glutamine (5 g) provided for 6 weeks.	Prebiotic intervention modified the microbiota structure Drastic decrease in alpha diversity parameters, as well as a change of beta diversity, without a clear directionality toward a healthy microbiota. * Sample size, study design

**Table 2:** Probiotics: Summary of selected studies.

Reference(s)	Study Design	Probiotics	Objectives	Probiotic Administration	Mains Results and Limitations *
Madhwani et al. (2011) [41]	In vitro	*L. reuteri ATCC 55730* *L. reuteri ATCC PTA 5289*	Investigation of the effects of an oral probiotic bacterium, *Lactobacillus reuteri* on the composition of nascent plaques (grown in short-term hydroxyapatite disc models) and in steady-state, continuous culture, in vitro dental plaques. Determination of the ecological fate of the probiotic bacterium in continuous culture in vitro plaques.	Not relevant	The introduction of *L. reuteri* bacteria to in vitro oral models resulted in alterations in both nascent and developed plaque ecosystems, which included increases in numbers of exogenous lactobacilli but also in increases in streptococci and Gram-negative anaerobes. *L. reuteri* bacteria persisted and potentially integrated into continuous culture dental plaque biofilms for at least 20 days following cessation of dosing. * In vitro study
Jiang et al. (2016) [42]	In vitro	*L. rhamnosus GG*	Investigation of the ability of probiotic *Lactobacillus rhamnosus GG* to integrate in oral biofilm and affect its species composition.	Not relevant	*L. rhamnosus* lowered the biofilm-forming ability of *F. nucleatum* and successfully integrated in all oral biofilms. *L. rhamnosus* reduced the counts of *S. sanguinis* and *C. albicans* *L. rhamnosus* only slightly reduced the adhesion of *S. mutans*. *C. albicans* significantly promoted the growth of L.GG. * In vitro study
Mendi et al. (2016) [43]	In vitro	*L. rhamnosus ATCC9595*	Determination of *P. gingivalis* modulatory effects on the inflammatory response of gingival stromal stem cells (G-MSSCs), including the release of CXCL8, and the expression of TLRs Determination of the *L. rhamnosus* preventive effect on CXCL8 inhibition in experimental inflammation.	Not relevant	*L. rhamnosus* showed higher adhesive properties than *P. gingivalis* on G-MSSCs. When G-MSSCs were pretreated with *L. rhamnosus* before *P. gingivalis* stimulation, CXCL8 secretions were found to increase. * In vitro study
Albuquerque-Souza et al. (2019) [44]	In vitro	*L. reuteri DSM 17938*, *L. rhamnosus Lr-32*™, *L. rhamnosus HN001*™, *L. acidophilus LA-5*™, *L. acidophilus NCFM*®, *L. casei 324 m*, *Bifidobacterium longum subsp. infantis ATCC15697*, *B. animalis subsp. lactis BB-12*™, *B. breve 1101A*, *B. longum 51A*, *B. pseudolongum 1191A*, *B. bifidum*	Evaluation of the effect of several clinical isolates and commercially available *Lactobacillus* sp. and *Bifidobacterium* sp. On gingival epithelial cells (GECs) challenged by *P. gingivalis*.	Not relevant	Probiotics may prevent cell death and reduce bacterial adhesion and invasion by *P.gingivalis*. Probiotics can modulate the inflammatory response mediated by *P. gingivalis* in GECs IL-1β and TNF-α synthesis stimulation decreases when co-culture with *P. gingivalis* and *L. rhamnosus* or bifidocteria (*B.longum*, *B. animalis*, *B. pseudolongum*, *B. bifidum*) or *L. salivarius*. CXCL8 secretion increases when co-culture with *P. gingivalis* and *L. salivarius* or *L. rhamnosus*. * In vitro study
Jaffar et al. (2016) [45]	In vitro	*L. acidophilus JCM*, *L. casei subsp. rhamnosus NBRC 3831*, *L. delbrueckii subsp. casei JCM 1012*, *L. fermentum JCM 1137*, *L. fermentum NBRC 15885*, *Lactococcus lactis NBRC 12007*, *L. casei NBRC 15883*, *Leuconostoc fructosum NBRC 3516*, *Leuconostoc mesenteroides IAM 1046*, *L. plantarum NBRC 15891*, *L. johnsonii NBRC 13952*, *L. sake NBRC 3541*, *L. paracasei subsp. paracasei NBRC 3533*	Evaluation of the potential of probiotic bacteria as a degrading agent against the periodontal pathogen *A. actinomycetemcomitans* and elucidation of the mechanisms underlying the observations made.	Not relevant	Probiotic bacteria demonstrate a robust degradation activity on *A. actinomycetemcomitans* Y4 and OMZ 534 strain, and a moderate effect against SUNY 75 strain Lipase enzyme from probiotic strains might be an influential factor in the biofilm degradation against *A. actinomycetemcomitans* Y4 and OMZ 534 strains. * No measurement of biofilm viability, In vitro study
Oliveira et al. (2017) [46]	Experimental study in rats (*n* = 32; 8 controls without EP, 8 EP only, 8 control + probiotic, 8 EP + probiotic group)	*Bifidobacterium animalis subsp. lactis HN019*	Evaluation of effects of topical administration of probiotic bacteria of the genus Bifidobacterium on experimental periodontitis (EP) in rats	Subgingival irrigation with 1 mL of suspension containing 1.9 × 10^9^ CFU of *B. lactis* HN019 on days 0, 3, and 7.	Compared with group EP, group EP-HN019 presented lower proportions of some Gram-negative anaerobic bacteria-like species involved in the pathogenesis of periodontal diseases Group EP-HN019 presented levels of IL-1b and an IL-1b/IL-10 ratio that were significantly reduced compared with group EP. * No identification of the subgingival microbiota before EP.
Invernici et al. (2020) [47]	randomized placebo-controlled study (*n* = 30, 15 Scaling root planning + placebo and 15 Scaling root planning + probiotic)	*Bifidobacterium animalis subsp. lactis HN019*	Evaluation of the effects of *B. lactis HN019* in non-surgical periodontal therapy in generalized chronic periodontitis patients. Investigation of the oral epithelial cell adhesion and antimicrobial properties of *B. lactis HN019*.	Lozenge (10^9^ CFU) intake 30 days after non-surgical periodontal therapy.	*B. lactis HN019* reduced the adhesion of *P. gingivalis* to buccal epithelial cells. *B. lactis* HN019 inhibit the growth of *P. gingivalis*, *P. intermedia*, *F. nucleatum* and *A. actinomycetemcomitans* in in vitro sensitivity tests. * Sample size
Dassi et al. (2014) [48]	placebo-controlled, parallel study (*n* = 12; 5 in control group, 7 in probiotic group)	*Lactobacillus delbrueckii subsp. bulgaricus*, *Lactobacillus paracasei*	Assessment of the impact on the overall saliva microbiome structure of a short-term probiotic intervention	100 g of a commercial probiotic product containing milk fermented (1 day).	Short-term probiotic intake significantly increases the complexity of the community with Steptococcus and Actinomyces as the most involved genera. Absence of significant changes detected in the metabolic structure of probiotic versus control samples. The two Lactobacillus strains present in the probiotic product were not detected in probiotic-intake samples. * Short probiotic intake, Sample size (*n* = 12)
Dassi et al. (2018) [49]	Cross sectional study (*n* = 21)	*Lactobacillus delbrueckii subsp. bulgaricus*, *Lactobacillus paracasei*	Verification of the hypothesis that the intake of commercially available probiotic products has a directly effect on the diversity and composition of the saliva microbiome.	100 g of a commercial probiotic product containing milk fermented (1 day).	The intake of commercially available probiotic products has a directly effect on the diversity and composition of the saliva microbiome, at least at short timescales. The overall taxonomic and abundance distribution of bacterial genera is however minimally influenced by probiotic intake. * Study design, sample size
Becirovic et al. (2018) [50]	Cross sectional study (*n* = 60)	*Lactobacillus acidophilus La-5*, *Bifidobacterium Bb-12*, *Lactobacillus rhamnosus GG*	Assessment of the effect of daily intake of a probiotic milk drink on the composition of bacterial species in dental supra- and subgingival biofilms.	200 mL probiotic milk beverage each day during 3 weeks	Influence of probiotics on bacteria in subgingival plaque was less than in supragingival plaque. The probiotic has led to a decrease in bacteria *Aggregatibacter actinomycetemcomitans*, *Actinomyces israelii*, *Actinomyces viscosus*, *Campylobacter rectus*, *Eikenella corrodens*, *Eubacterium saburreum*, *Fusobacterium nucleatum ssp. nucleatum*, *Porphyromonas endodontalis*, *Prevotella intermedia*, *Porphyromonas gingivalis*, *Parvimonas micra*, *Prevotella nigrescens*, *Streptococcus intermedius*, *Treponema denticola*, *Tannerella forsythia* in supragingival plaque. In the subgingival plaque a decrease of *Actinomyces viscosus*, *Fusobacterium nucleatum ssp. nucleatum*, Treponema socranskii ssp. Socranskii * Study design, sample size
Tada et al. (2018) [51]	Randomized placebo-controlled study (*n* = 30; 15 placebo group, 15 probiotic group)	*L. reuteri DSM 17938*, *L. reuteri ATCC PTA 5289*	Investigation of the effects of a probiotic tablet containing *Lactobacillus reuteri* in peri-implantitis patients.	One tablet a day for 6 months with 1 × 10^8^ CFU *L. reuteri strains DSM 17938* and *ATCC PTA 5289*.	Negligible changes were observed in the bacterial flora around implants * Sample size, No evaluation of *L. reuteri* colonization.
Kõll et al. (2008) [52]	In vitro	*L. acidophilus*, *L. crispatus*, *L. delbrueckii*, *L. gasseri*, *L. salivarius*, *L. paracasei*, *L. plantarum*, *L. rhamnosus*, *L. fermentum*, *L. oris*	Characterization of oral lactobacilli for their potential probiotic properties according to the international guidelines for the evaluation of probiotics. Selection of oral lactobacilli strains that could eventually be used as probiotics for oral health.	Not relevant	Several human oral lactobacilli possess good functional probiotic properties like antimicrobial activity against oral pathogens as well as high tolerance of environmental stress factors. These beneficial properties are better expressed in *L. plantarum*, *L. paracasei*, and *L. rhamnosus*, *L. salivarius* strains. The strains of *L. plantarum* differ from the natural resistance pattern of lactobacilli and therefore, should be considered non-safe * In vitro study
Teanpaisan et al. (2011) [53]	In vitro	*L. paracasei*, *L casei*, *L. salivarius*, *L. plantarum*, *L. rhamnosus*, *L. fermentum*, *L. gasseri*, *L.mucosae*, *L. oris*, *L. vaginalis*	Determination of the inhibitory effect of oral Lactobacillus against putative oral pathogens.	Not relevant	*L. paracasei*, *L. casei*, *L. salivarius*, *L. plantarum*, *L. rhamnosus*, *L. fermentum* have a strong inhibitory effect against *S. mutans* and *Streptococcus sobrinus*, as well as, Gram-negative periodontal pathogens *Porphyromonas gingivalis* and *Aggregatibacter actinomycetemcomitans*. * In vitro study
Van Essche et al. (2013) [54]	In vitro	*L. rhamnosus*, *L. casei*, *L. fermentum*, *L. paracasei*	Assessment of the antagonistic potential of oral bacteria on periodontal pathogens. Evaluation of the inhibitory effect of some commercial dietary probiotics on periodontopathogens and comparison with the inhibitory effect of orally derived beneficial bacteria.	Not relevant	The commensal oral microbiota is considered to induce a beneficial oral immune response or to interfere with periodontopathogen colonization. Probiotics showed a stronger inhibition of *P. gingivalis* and *P. intermedia*, and the oral isolated strains showed a clearly stronger inhibition of *F. nucleatum* and *A. actinomycetemcomitans*. * In vitro study
Chen et al. (2020) [55]	In vitro	*L.salivarius subsp. salicinius AP-32*, *L. rhamnosus CT-53*, *L. paracasei ET-66*, *Bifidobacterium animalis subsp. lactis CP-9*, *L. acidophilus TYCA02*	Evaluation of the antipathogenic efficacy of different probiotic species and their potential roles in developing functional foods to improve oral health	Not relevant	*Lactobacillus salivarius subsp. salicinius AP-32*, *L. rhamnosus CT-53*, *L. paracasei ET-66* and *B. animalis subsp. lactis CP-9* displayed strong antibacterial activity against the oral pathogens *S. mutans*, *P. gingivalis*, *F. nucleatum* and *A. actinomycetemcomitans*. * In vitro study
Shin et al. (2018) [56]	In vitro	*Lactococcus lactis HY449*	Investigation of the inhibitory effects of *L. lactis* on the bioactivity of periodontopathogens.	Not relevant	*L. lactis* has antimicrobial activity against periodontopathogens, such as *F. nucleatum*, *P. gingivalis*, *T. forsythia*, and *T. denticola*. *L. lactis* neutralized and inhibited inflammatory cytokines induced by lipopolysaccharides derived from these pathogens. * In vitro study
Higuchi et al. (2019) [57]	In vitro	*Lactobacillus salivarius WB21* combined with green tea catechins	Evaluation of the combined use of *Lactobacillus salivarius WB21* and epigallocatechin gallate for oral health maintenance.	Not relevant	Growth of *P. gingivalis* was strongly inhibited by co-culture with *L. salivarius WB21* combined with green tea catechins * In vitro study
Zhu et al. (2010) [58]	In vitro	*L. bulgaricus*, *S. thermophilus*, *L. acidophilus*, *Bifidobacterium*	Investigation of the competition between probiotics and periodontal pathogens in vitro.	Not relevant	Probiotics are capable of inhibiting specific periodontal pathogens but have no effect on the periodontal protective bacteria (*S. sanguinis*). Competition between probiotics and periodontal pathogens depended on the sequence of inoculation. * In vitro study
Tobita et al. (2018) [59]	Randomized double blind placebo-controlled study (*n* = 16; 8 control, 8 probiotic group)	*L. crispatus KT-11* (KT11)	Examination of the effects of KT-11 consumption on the oral environment in healthy volunteers.	A KT-11 food tablet (1.2 × 10^10^ KT-11 cells) every day for 4 weeks	Daily KT-11 intake can prevent periodontal disease through the improvement of oral conditions. KT-11 makes *P. gingivalis* numbers decrease. * Sample size
Iniesta et al. (2012) [60]	placebo-controlled, parallel study (*n* = 40; 20 control, 20 probiotic group)	*L. reuteri ATCC-PTA-5289*, *L. reuteri DSM-17938*	Investigation of the effects of an orally administered probiotic on the oral microbiota.	one tablet per day, during 28 days One tablet per day, during 28 days with *L. reuteri DSM-17938* and *ATCC PTA5289* at a dose of 2 × 10^8^CFU/tablet.	*L. reuteri* administered in tablets resulted in a reduction in the number of *Prevotella intermedia* and *Porphyromonas gingivalis* in the subgingival microbiota, without an associated clinical impact. *L. reuteri* containing probiotic tablets are able to colonize the saliva and the subgingival habitat. * Differences between groups that, although not significant, may have influenced the outcomes, short time evaluation (follow up 8 weeks)
Teughels et al. (2017) [61]	Randomized placebo-controlled study (*n* = 30; 15 control, 15 probiotic group)	*L. reuteri DSM17938*, *L. reuteri ATCC PTA-5289*	Evaluation of the effects of *L. reuteri* as an adjunct to scaling and root planning.	One lozenge at the morning and one at the night during 12 weeks (*L. reuteri* 1 × 10^8^ CFU for each strain)	*L. reuteri* lozenges resulted in significant additional clinical improvements primarily for initially moderate to deep pockets when compared to SRP alone. The microbiological differences were more moderate and primarily restricted to P. gingivalis numbers. * Sample size, No evaluation of *L. reuteri* colonization.
Montero et al. (2015) [62]	Randomized double blind placebo-controlled study (*n* = 59 patients; 29 tests, 30 placebos)	*L. plantarum*, *L. brevis*, *P. acidilactici*	Evaluation of the efficacy of a probiotic combination in the treatment of gingivitis Assessments of the impact of a probiotic combination on the subgingival microbiota	One lozenge at the morning and one at the night during 12 weeks (*L. reuteri* 1 × 10^3^ CFU for each strain)	Use of probiotic tablets containing *L. plantarum*, *L. brevis* and *P. acidilactici* did not lead to significant changes in mean gingival index; although a significant reduction occurred in the number of sites with severe inflammation. The adjunctive use of the probiotic also demonstrated a significant microbiological impact by reducing the counts of *T. forsythia*.
Imran et al. (2015) [63]	Cross sectional study (*n* = 42)	*L. casei Shirota*	Evaluation of the impact of Probiotic drink containing *Lactobacillus casei Shirota* on the bacterial population in subgingival plaque in patient with chronic generalized mild to moderate periodontitis.	65 mL of probiotic milk (Yakult©) once daly for one month (10^8^ CFU/mL of *L. casei* strain Shirota).	Oral administration of the probiotic lactobacilli reduced the numerical sum of *A. actinomycetemcomitans*, *P. intermedia*, *P. gingivalis*. No statistically significant changes were observed in the gingival index and plaque index scores. * Sample size
Sarmento et al. (2019) [64]	Randomized placebo-controlled study (*n* = 41; 20 control group, 21 probiotic group)	*L. casei*	Evaluation of the effect of petit-Suisse plus probiotic on the microbiota of children’s saliva.	50g of petit-suisse cheese daily from Monday to Friday, and the following week from Monday to Thursday (3% de *L. casei*)	The product with added *L. casei* was shown to be able to reduce *A. actinomycetemcomitans*, and able to maintain lower density of *P. gingivalis* in post treatment two weeks later. * No evaluation of probiotic change after introduction in food.
Mayanagi et al. (2009) [65]	Randomized placebo-controlled study (*n* = 36; 32 control group, 34 probiotic group)	*L. salivarius WB21*	Evaluation of the impact of oral administration of lactobacilli on the bacterial population in supra/subgingival plaque.	One tablet containing *L. salivarius WB21* (6.7 × 10^8^ CFU/tab) and xylitol (280 mg/tab) three times per day during 8 weeks.	*L. salivarius WB21* administration successfully decreased the numerical sum of *A. actinomycetemcomitans*, *P. intermedia*, *P. gingivalis*, *T. denticola*, and *T. forsythia* in subgingival plaque at 4 weeks. No significant difference between the WB21 and the placebo groups in the direct count of any specific periodontopathic bacteria at 8 weeks. * Sample size
Sajedinejad et al. (2018) [66]	Randomized placebo-controlled study (*n* = 50; 50 control group, 50 probiotic group)	*Lactobacillus salivarius NK02*	Evaluation of the effects of probiotic mouthwash and scaling and root planning (SRP) on clinical and microbiological parameters of moderate to severe periodontitis.	Bottle of 20 mL of mouthwash was used twice a day after brushing the teeth for 28 days (10^8^ CFU/mL of *L. salivarius NK02*)	Probiotic mouthwash was able to inhibit the bacterial growth on both saliva and sub-gingival crevice and exhibited antibacterial activity against *A. actinomycetemcomitans*. * No long term follow up
Zahradnik et al. (2009) [67]	Cross sectional pilot study (*n* = 12)	*S. oralis KJ3sm*, *S. uberis KJ2sm*, *S. rattus JH145*	Test the ability of a probiotic mouthwash, ProBiora3, to affect the levels of *Streptococcus mutans* and certain known periodontal pathogens in the mouth.	Bottle of 20 mL of mouthwash was used twice a day after brushing the teeth for 28 days (10^8^ CFU/mL of *S. oralis KJ3sm*, *S. uberis KJ2sm*, *S. rattus JH145*).	The probiotic mouthwash was able to substantially affect the levels of dental pathogens in saliva (*S.mutans*) and periodontal pathogens in subgingival plaque (*C. rectus* and *P.gingivalis*) * Sample size, Young and orally healthy adults poupulation
Alanzi et al. (2018) [68]	Randomized placebo-controlled study (*n* = 108, 54 placebo group, 54 probiotic group)	*L. rhamnosus GG*, *B lactis BB12*	Determination of the effect of a probiotic combination on the gingival health, dental plaque accumulation, and the oral carriage of four putative periodontal pathogens in healthy adolescents	Lozenges twice a day during a four-week period (probiotic lozenge contained LGG 4.4 × 10^8^ CFU and BB-12 4.8 × 10^8^ CFU)	The short-term daily consumption of LGG and BB-12 probiotic lozenges improved the gingival health in adolescents The short-term daily consumption of LGG and BB-12 probiotic lozenges decreased levels of *A. actinomycetemcomitans* and *F. nucleatum* both in saliva and plaque and decreased *P. gingivalis* count in plaque. * Sample size, short term evaluation
Morales et al. (2018) [69]	Randomized placebo-controlled study (*n* = 47, 16 SRP + probiotic, 16 SRP + antibiotic, 15 SRP + placebo)	*L. rhamnosus SP1*	Evaluation of the effects of *Lactobacillus rhamnosus SP1*-containing probiotic sachet and azithromycin tablets as an adjunct to nonsurgical therapy in clinical parameters and in presence and levels of *Tannerella forsythia*, *Porphyromonas gingivalis* and *Aggregatibacter actinomycetemcomitans*.	1 sachet in water (150 mL) ingested once a day after brushing their teeth (2 × 10^7^ CFU/day)	All groups showed improvements in clinical and microbiological parameters at all time points evaluated. Probiotic and antibiotic groups showed greater reductions in cultivable microbiota compared with baseline. The placebo group showed greater reduction in number of subjects with *P. gingivalis* compared with baseline. However, there were no significant differences between groups The adjunctive use of *L. rhamnosus SP1* sachets and azithromycin during initial therapy resulted in similar clinical and microbiological improvements compared with the placebo group. * Sample size
Kobayashi et al. (2017) [70]	Experimental study in mice (*n* = 108; 36 tréhalose + Pg, 36 LG2055 + Pg, 36 placebo group)	*L. gasseri SBT2055*	Assessment of the potential of oral administration of *Lactobacillus gasseri SBT2055* (LG2055) for *Porphyromonas gingivalis* infection	Oral intubation with a LG2055 suspension (1 × 10^9^ CFU/200 µL/mouse) through a syringe fitted with a ball-type feeding needle once per day for 5 weeks.	LG2055 treatment significantly reduced alveolar bone loss, detachment and disorganization of the periodontal ligament, and bacterial colonization by subsequent *P. gingivalis* challenge. The expression and secretion of TNF-α and IL-6 in gingival tissue was significantly decreased in LG2055-administered mice after bacterial infection. * Experimental design
Maekawa et al. (2014) [71]	Experimental study in mice (*n* = 6; 3 probiotic, 3 placebo group)	*L. brevis CD2*	Determination of the *Lactobacillus brevis CD2* potential on inhibit periodontal inflammation and bone loss in experimental periodontitis.	Lyopatch with *L. brevis CD2* (8 × 10^5^ CFU in 1-mm^2^)	*L. brevis CD2*-treated mice exhibited significantly decreased expression of all proinflammatory cytokines tested (TNF, IL-1β, IL-6, and IL-17A). *L. brevis CD2* treatment resulted in significantly higher counts of aerobic bacteria and, conversely, significantly lower numbers of anaerobic bacteria, as compared to the placebo-treated control group. * Sample size
Keller et al. (2018) [72]	Randomized placebo-controlled study (*n* = 47, 23 probiotic, 24 placebo group)	*L. rhamnosus PB01*, *L. curvatus EB10*	Investigation of the clinical and the microbial effects of probiotic candidate strains in patients with moderate gingivitis.	One tablet in the morning and one in the evening 30 min after tooth brushing containing a mix of *L. rhamnosus* and *L. curvatus* (10^8^ CFU/tablet)	The concentration of selected cytokines (interleukin (IL 1β, IL6, IL8, IL10, tumour necrosis factor alpha (TNF-α)) in gingival crevicular fluid were unaffected by the intervention as well as the salivary microbiome. * Sample size, healthy patient
Braathen et al. (2017) [73]	randomised, double-blind, placebo-controlled, cross-over trial (*n* = 47)	*L. reuteri DSM 17938*, *L. reuteri ATCC PTA 5289*	Comparaison of the concentration of salivary immunoglobulin A (IgA) and the selected interleukins (IL)-1β, IL-6, IL-8 and IL-10 in young individuals with presence and non-presence of *Lactobacillus reuteri* in saliva after a three-week intervention with probiotic lozenges.	Two lozenges per day containing the probiotic bacterium *L. reuteri* for three weeks (L. reuteri DSM 17938 1 × 10^9^ *CFU/lozenge*, *ATCC PTA5289* 2 × 10^9^ CFU/lozenge)	No differences in the cytokine levels (IL1β, IL-6, IL-8 and IL-10) were observed. Individuals with presence of *L. reuteri* in saliva had significantly higher concentrations of salivary IgA * Sample size, healthy patient
Jørgensen et al. (2016) [74]	randomised, double-blind, placebo-controlled, cross-over trial (*n* = 47)	*L. reuteri DSM 17938*, *L. reuteri ATCC PTA 5289*	Evaluation of the effect of daily ingestion of probiotic lactobacilli on the levels of secretory IgA (sIgA) and selected cytokines in whole saliva of healthy young adults.	Two lozenges per day containing *L. reuteri DSM 17938*, *L. reuteri ATCC PTA 5289* *L. reuteri DSM 17938*, *L. reuteri ATCC PTA 5289*	No significant differences in the concentrations of salivary sIgA or cytokines were recorded between the *L. reuteri* and placebo interventions or between baseline and 3 weeks post-intervention levels. * Sample size, healthy patient
Hallström et al. (2016) [75]	Randomized placebo-controlled study. (*n* = 46; 22 probiotic, 24 placebo group)	*L. reuteri* DSM 17938, *L. reuteri* ATCC PTA 5289	Evaluation of the effects of probiotic supplements in adjunct to conventional management of peri-implant mucositis	Topical oil application (2 × 10^7^ CFU of each strain) followed by twice-daily intake of lozenges for 3 months (1 × 10^8^ CFU of each strain)	Topical treatment and daily intake of probiotic lozenges as an adjunct to mechanical debridement and oral hygiene instructions did not improve clinical, microbial or inflammatory variables of peri implant mucositis as compared to the use of placebo. * Sample size

CFU: colony forming units.

**Table 3:** Probiotics involved in modulating oral bacterial species associated with mental disorders.

Lactobacilli	Bifidobacterium	Streptococci	Others *
*L. rhamnosus*	*B. animalis*	*S. thermophilus*	*L. lactis*
*L. reuteri*	*B. longum*	*S. uberis*	*P. acidilactici*
*L. salivarius*	*B. pseudolongum*	*S. rattus*	
*L. caseï*		*S. oralis*	
*L. paracaseï*			
*L. delbruekii*			
*L. acidophilus*			
*L. plantarum*			
*L. fermentum*			
*L. gasseri*			
*L. crispatus*			
*L. brevis*			
*L. curvatus*			

* *L. lactis*: *Lactococcus lactis*; *P. acidilactici*: *Pediococcus acidilactici*.

## Data Availability

Not applicable.
